# The influence of smoking and HIV infection on pulmonary function

**DOI:** 10.4102/sajhivmed.v23i1.1329

**Published:** 2022-02-21

**Authors:** Annelotte E. Sussenbach, Sjors W.L. van Gijzel, Samanta T. Lalla-Edward, Willem D.F. Venter, Erica Shaddock, Charles Feldman, Kerstin Klipstein-Grobusch, Alinda G. Vos

**Affiliations:** 1 Julius Global Health, Julius Center for Health Sciences and Primary Care, University Medical Center Utrecht, Utrecht, the Netherlands; 2 Ezintsha, Faculty of Health Sciences, University of the Witwatersrand, Johannesburg, South Africa; 3 Division of Pulmonology, Department of Internal Medicine, Charlotte Maxeke Johannesburg Academic Hospital, Johannesburg, South Africa; 4 Division of Pulmonology, Faculty of Health Sciences, University of the Witwatersrand, Johannesburg, South Africa; 5 Division of Epidemiology and Biostatistics, School of Public Health, Faculty of Health Sciences, University of the Witwatersrand, Johannesburg, South Africa

**Keywords:** HIV, lung function, PLWH, South Africa, spirometry, sub-Saharan Africa

## Abstract

**Background:**

Prevalence of HIV, smoking, and pulmonary infections in South Africa are high.

**Objectives:**

We investigated the role of smoking and HIV status on lung function.

**Methods:**

This is a secondary analysis of a cross-sectional study conducted in South Africa. Data included demographics, pulmonary risk factors and a spirometry test to obtain the forced expiratory volume in one second (FEV1) and the ratio of FEV1/forced vital capacity (FVC). In the initial multivariable regression analysis, the effect of smoking on pulmonary function in HIV-positive adults was assessed. The analysis was repeated, assessing the influence of HIV status on lung function in both HIV-negative and HIV-positive smokers. The models were adjusted for age, sex, body mass index (BMI), time since HIV diagnosis, antiretroviral treatment (ART) use, occupational hazards, history of tuberculosis or pneumonia, indoor smoking and the presence of an indoor fireplace during childhood.

**Results:**

This study included 524 people living with HIV (PLWH, 66.7% female, mean age 40.9 years [s.d.; 9.4]) and 79 HIV-negative smokers (77.2% male, mean age 34.4 years [s.d.: 12.1]). Of the PLWH, 118 (22.5%) were past or current smokers and 406 (77.5%) were non-smokers. Smoking was not associated with changes in the FEV1 or FEV1/FVC ratio in multivariable regression analysis. In the second analysis, HIV status was also not associated with reduced pulmonary function following adjustment for confounders.

**Conclusion:**

Neither smoking nor being HIV-positive was associated with decreased pulmonary function in this relatively young population. These findings should be confirmed in a longitudinal study, including an older population.

## Introduction

HIV has infected over 75 million people, with over 32 million deaths reported worldwide since its emergence in the 1980s.^1^ Even though there is no cure for HIV infection, the use of antiretroviral treatment (ART) has resulted in a near normal life expectancy for people living with HIV (PLWH).^2,3^

This increased life expectancy has resulted in an increase in age-related comorbid diseases, such as cardiovascular diseases, diabetes mellitus and cancers.^2,3^ Untreated HIV is associated with an increased vulnerability to pulmonary infections, such as pneumonia and pulmonary tuberculosis (TB), resulting from the immune dysfunction caused by the virus.^2,4,5,6^ Although ART restores immune function in PLWH, immunity does not return to normal levels and chronic immune activation persists. This low-grade immune activation is also manifested in the immune cells that reside in the lungs and may be responsible for HIV-related lung damage.^7^ Furthermore, ART and HIV might be independently associated with a decline in pulmonary function.^8,9^

Although smoking rates are decreasing globally, about 80% of smokers live in low- and middle-income countries (LMICs), which also have the highest prevalence of HIV.^1,10,11^ In high-income countries, the incidence of smoking amongst PLWH is still two to three times higher than in people without HIV.^12^ The prevalence of smoking amongst PLWH in sub-Saharan Africa ranges from lower to higher than the HIV-uninfected community.^13,14,15,16,17,18,19,20,21^ In South Africa, the prevalence of smoking in PLWH is estimated to be around 20%.^13,16^ Although rates differ between provinces and different social groups, these numbers are concerning, given the background of pre-existing lung damage because of (opportunistic) infections and the possible negative effects of HIV and ART on lung function.

Many studies have investigated the added negative effect of smoking on pulmonary function in PLWH.^4,8,22,23,24,25^ The majority of these cross-sectional studies reported that the negative impact of smoking on lung function in PLWH could not be attributed to a history of pulmonary infections, such as TB and pneumonia alone. Therefore, this study aimed to investigate the influence of smoking and HIV infection on pulmonary function in Johannesburg, South Africa.

## Methods

Data from two existing study databases were combined in this analysis.

### Study population and data collection

Study Group I: The aim of Study I was to investigate the interaction between HIV, ART, pulmonary function, and cardiovascular traits in an urban South African population. The inclusion criteria for this study were as follows: (1) confirmed HIV-positive status, age ≥ 18 years and enrolled at the HIV clinic at the Charlotte Maxeke Johannesburg Academic Hospital (CMJAH); (2) individuals aged ≥ 18 years with unknown HIV status who participated in the Universal Test and Treat (UTT) programme at CMJAH and who agreed to share their test result with the researcher. The South African UTT initiative was founded in 2016 and aimed to test as many people as possible, and to initiate ART regardless of the CD4 count in those testing positive for HIV.^26,27^

Participants who are HIV negative were enrolled through the UTT programme. Participants unable to undergo study procedures for any reason or unwilling to share HIV test results or with unknown HIV status were excluded from the study. A written informed consent was obtained from all study participants.

Study Group II: This study aimed to investigate the interaction between HIV, ART and cardiopulmonary function in an urban South African population. Study II was also conducted at CMJAH and included PLWH not yet on ART, well-characterised PLWH on first- and second-line ART, as well as HIV-negative controls as previously described.^28^ Participants were recruited from randomised controlled trials (RCTs) comparing ART regimens. Inclusion and exclusion criteria included those of Study I, and additionally excluded pregnant women, people with impaired kidney function and people on active TB treatment. The methods are described in more detail in previous publications.^15,29^

In both studies, a spirometry test was performed, next to questionnaires on participants’ demographics, characteristics and respiratory profile. The respiratory questionnaire included The St George’s Respiratory Questionnaire (SGRQ).^30^ Spirometry was performed with a handheld device, and included pre- and post-bronchodilator measurements. Acceptability and reproducibly were based on guidelines of the American Thoracic Society and European Respiratory Society.^31,32^ More details on measurements can be found in the supplementary file (Table 1-A1).

A written informed consent was obtained from all study participants. REDCap version 9.7.8 was used for data collection in both studies.^39,40^

### Data analysis

Baseline characteristics of participants were presented as mean with standard deviation (s.d.) or as frequencies and percentages, unless stated otherwise. The characteristics were compared using a *t*-test for continuous variables and the Chi-square test for categorical variables.

The primary outcome of this study was post-bronchodilator FEV1. The secondary outcome was the post-bronchodilator FEV1/forced vital capacity (FVC) ratio.

Multivariable regression analyses were performed to test the relationship between smoking and pulmonary function in PLWH. The following variables were regarded as confounders based on the literature and were included in the models using forced entry of variables: age, sex, body mass index (BMI), time since HIV diagnosis, ART duration, history of pneumonia, occupational hazard, indoor smoking in the household and presence of a fireplace during childhood.^9,23,41^ The first model assessed the effect of smoking on post-bronchodilator FEV1, whilst the second model was corrected for the above-mentioned covariates. In a third model, a history of TB was added to account for possible effect modification. All steps were repeated using the FEV1/FVC ratio as outcome.

In an additional analysis, smoking was treated as a continuous variable using pack-years, with zero pack-years for non-smokers (information on pack-years was only available for Study II).

In a second analysis, the influence of HIV on lung function in smokers was assessed using the same steps as described above. HIV status was the main determinant, and the same confounders were taken into account, including smoking status, except for the HIV-related variables, including time since HIV diagnosis and ART duration.

Multicollinearity was checked for all variables in the models.^42^ A two-sided *P*-value of < 0.05 was considered to be statistically significant. Initially, complete case analysis was performed. Subsequently, missing data were imputed using multiple imputation with the R-package MICE.^43,44^ R version 4.0.0 and Statistical Package for the Social Sciences (SPSS) version 26 were used for data analysis.^45,46^

### Ethical considerations

All participants were required to sign a written informed consent form prior to enrolment. In case the participant was not able to read the English form, an additional consent form had to be signed that verbal explanation of the form was sufficient. The University of the Witwatersrand, Human Research Ethics Committee (HREC) approved Study I (M190628) and Study II (M160130).

## Results

**FIGURE 1:**
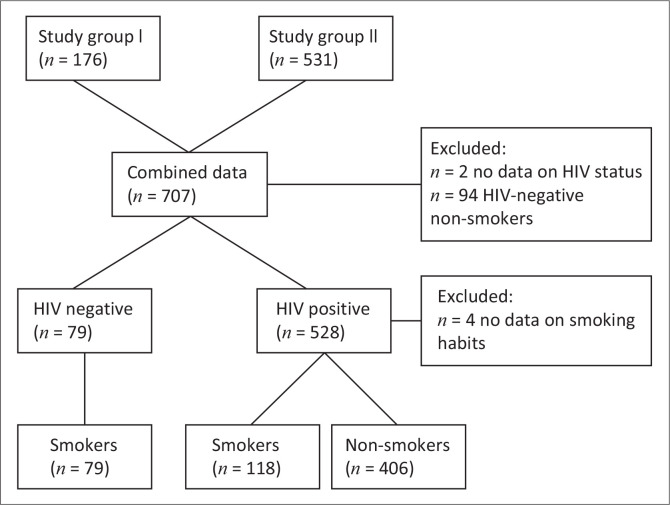
Flow diagram of study composition.

In total, 524 PLWH were included (66.7% female), of whom 118 individuals were past or current smokers (118/524 (22.5%) (Figure 1). Four were excluded from analyses because of missing information on the smoking status. The mean age was 40.9 years (s.d.: 9.5), the mean time from HIV diagnosis was 92.3 months (s.d.: 69.6) and the mean ART duration was 74.5 months (s.d.: 60.6). One-third of the participants had been previously diagnosed with TB (33.0%), and approximately 10% had been previously diagnosed with pneumonia.

Past or current smokers were more likely to be men; had a lower BMI; were less likely to be on ART; were more likely to consume alcohol; and were more likely to be exposed to chemicals, gases or fumes; working in a dusty job; or working in the farming or mining industry for more than a year (Table 1).

**TABLE 1:** Baseline characteristics of the study population stratified by smoking.

Characteristics	HIV-positive								HIV-negative: Past or current smokers			
	Non-smokers				Past or current smokers							
	*n*	%	Mean	s.d.	*n*	%	Mean	s.d.	*n*	%	Mean	s.d.
	406	-	-	-	118	-	-	-	79	-	-	-
Age (years)*	-	-	40.65	8.84	-	-	41.83	11.39	-	-	34.43	12.08
Non-black	1	0.2	-	-	3	2.5	-	-	8	10.1	-	-
Male	98	24.1	-	-	77	65.3	-	-	61	77.2	-	-
Time since the HIV diagnosis (months)*	93.25	67.75	-	-	87.55	74.13	-	-	-	-	-	-
HIV positive on ART	339	83.5	-	-	85	72.0	-	-	-	-	-	-
ART duration (months)*	-	-	76.85	58.54	-	-	69.02	66.64	-	-	-	-
Body mass index (kg/m^2^)*	-	-	27.72	6.61	-	-	25.65	5.91	-	-	23.70	5.09
Education: secondary completed or higher	350	87.1	-	-	94	80.3	-	-	73	93.6	-	-
**Work status**												
Employed	138	34.2	-	-	47	40.2	-	-	53	67.9	-	-
Unemployed	258	63.9	-	-	65	55.6	-	-	17	21.8	-	-
Retired	6	1.5	-	-	3	2.6	-	-	1	1.3	-	-
Student	2	0.5	-	-	2	1.7	-	-	7	9.0	-	-
In stable relationship†	143	35.4	-	-	40	34.2	-	-	15	19.0	-	-
Alcohol use†	184	45.4	-	-	97	83.6	-	-	65	82.3	-	-
Fire time [> daily]†	267	65.9	-	-	68	57.6	-	-	61	77.2	-	-
Smoke household†	75	18.7	-	-	31	27.0	-	-	18	22.8	-	-
Smoke household indoor†	51	12.7	-	-	17	14.8	-	-	11	14.5	-	-
Childhood fireplace†	268	66.0	-	-	87	74.4	-	-	57	73.1	-	-
Parental lung disease†	23	5.7	-	-	7	5.9	-	-	2	2.5	-	-
Occupational hazard†	40	9.9	-	-	27	22.9	-	-	5	6.3	-	-
History of tuberculosis	132	32.6	-	-	40	33.9	-	-	2	2.5	-	-
History of pneumonia	40	9.9	-	-	13	11.1	-	-	4	5.1	-	-
Asthma	28	6.9	-	-	7	6.0	-	-	5	6.3	-	-
Breathing problems†	16	4.0	-	-	10	8.5	-	-	6	7.7	-	-

† , Definitions of variables can be found in supplementary material (Table 1-A1).

Smoking was not significantly related to post-bronchodilator FEV1 or the FEV1/FVC ratio following regression analysis (Table 2). There was no relationship between FEV1 and time since HIV diagnosis or time on ART. Adding TB to the models did not alter the relationship between smoking and FEV1 or the FEV1/FVC ratio. Tuberculosis was associated with a significant decline in both the FEV1 and the FEV1/FVC ratio.

**TABLE 2:** Results of multivariable analysis for post-bronchodilator (BD) forced expiratory volume in 1 second and forced expiratory volume in 1 second/forced vital capacity on HIV-positive participants.

Variable	Model 1: without history of TB		Model 2: with history of TB	
	Post-BD FEV1 (β-coefficient†)	Post-BD FEV1/FVC (β-coefficient†)	Post-BD FEV1 (β-coefficient†)	Post-BD FEV1/FVC (β-coefficient†)
Smoking ever	–0.016	0.001	–0.012	0.002
Age (years)	–0.022**	–0.002**	–0.022**	–0.002**
Sex (male)	0.838**	–0.020**	0.848**	–0.002**
BMI (kg/m^2^)	–0.009*	–0.001*	–0.009**	–0.001*
Time since HIV diagnosis (months)	< 0.001	< 0.001	< 0.001	< 0.001
ART duration (months)	< –0.001	< –0.001	–0.001	< –0.001
Occupational hazard‡	0.058	<0.001	0.450	< –0.001
History of pneumonia	–0.142*	–0.022*	–0.099	–0.099
Smoke household indoor‡	–0.051	0.003	–0.046	0.004
Fireplace childhood‡	–0.121**	0.001	–0.122*	0.001
History of TB	-	-	–0.195**	–0.017**

TB, tuberculosis; BD: bronchodilator; BMI, body mass index; FEV1, forced expiratory volume in 1 second; FVC, forced vital capacity; ART, antiretroviral treatment.

* , *P* < 0.05;

** , *P* < 0.01.

† , β-Coefficients are the result of multivariable analysis and are unstandardised;

‡ , Definitions of variables can be found in Methods: Measurements.

Similar results were reported when smoking was quantified using pack-years (only available for Study II), that is, smoking not being significantly associated with FEV1 nor FEV1/FVC (data not shown). However, the Spearman correlation coefficient between pack-years and pulmonary function was *R* = 0.40 (*P* < 0.01).

In the second analysis, 197 smokers were analysed (71.1% part of Study II), of whom 118 were HIV-positive individuals (59.9%) (Table 1). The mean age was 38.9 years (s.d.: 12.2), with 70.0% being male participants. The mean smoking time was 15.2 years (s.d.: 0.7). HIV status was not significantly associated with pulmonary function following regression analysis. Adding TB to the model did not alter results. A history of TB was significantly associated with a decline in both outcomes (Table 3).

**TABLE 3:** Results of multivariable analysis for post-bronchodilator (BD) forced expiratory volume in 1 second and forced expiratory volume in 1 second/forced vital capacity on smoking participants.

Variable	Model 1: without history of TB		Model 2: with history of TB	
	Post-BD FEV1 (β-coefficient†)	Post-BD FEV1/FVC (β-coefficient†)	Post-BD FEV1 (β-coefficient†)	Post-BD FEV1/FVC (β-coefficient†)
HIV positive	–0.027	–0.027	0.025	0.025
Age (years)	–0.026**	–0.026**	–0.025**	–0.025**
Sex (male)	0.771**	0.771**	0.768**	0.768**
BMI (kg/m^2^)	–0.021*	–0.021*	–0.020*	–0.020*
Time since the HIV diagnosis (months)	< 0.001	< 0.001	0.0001	< 0.001
Occupational hazard‡	0.121	0.122	0.113	0.113
History of pneumonia	0.115	–0.115	0.195	0.195
Smoke household indoor‡	0.034	0.034	0.033	0.033
Fireplace childhood‡	–0.260*	–0.260*	–0.274*	–0.274*
History of TB	-	-	–0.224*	–0.224*

TB, tuberculosis; BD, bronchodilator; BMI, body mass index; FEV1, forced expiratory volume in 1 second; FVC, forced vital capacity.

* , *P* < 0.05;

** , *P* < 0.01.

† , *β*-Coefficients are the result of multivariable analysis and are unstandardised;

‡ , Definitions of variables can be found in Methods: Measurements.

Analyses based on data including multiple imputation for missing data showed comparable results (Supplementary Table 1-A1).

## Discussion

In this study of PLWH carried out in Johannesburg, South Africa, no relationship between smoking or HIV positivity and FEV1 or the FEV1/FVC ratio was observed. However, a history of TB was associated with a decrease in lung function.

The finding of this study that smoking was not associated with a decrease in pulmonary function in PLWH was unexpected, yet interestingly in line with other cross-sectional studies from high-income countries.^4,23,47^ In most studies, a decline in pulmonary function was explained by effects of HIV, or by irreversible pulmonary damage because of TB or pneumonia, and not by smoking. However, one study from Pittsburgh, United States, observed that smoking was significantly associated with diffusion impairment in PLWH.^8^ Cross-sectional studies from sub-Saharan Africa also found that smoking was not associated with decreased pulmonary function.^9,24^ In general, the average age of the study populations was around 50 years. We found that age was significantly associated with a decrease in lung function, suggesting that the deleterious effects of smoking on lung function may only become apparent with increasing age. Studies that did report any adverse effect of smoking on lung function often limited their inclusion to individuals over 40 years of age.^23,24,25^

The prevalence of ever smoking was found to be 22% in this study, in line with other studies from South Africa and other sub-Saharan African countries.^12,48^ However, studies have reported a higher prevalence of smoking in PLWH (34% – 37%^13,15,19,49^) than in the general population in South Africa, despite various cessation strategies implemented by the government.^20,50,51^ Other studies in sub-Saharan Africa reported a prevalence of smoking in HIV-positive men ranging from 9.7% in Ethiopia to 54.8% in the Gambia.^17,18,19,20,21^ In the current study, there was no validation of self-reported smoking, whilst in most studies mentioned above, validation of current smoking was carried out by testing of urine samples for cotinine. Inadequate reporting of smoking could be a possible explanation for the lower prevalence of smoking in this study. Another explanation might be that in this study, there was a relatively lower prevalence reported from a single centre (CMJAH) in Johannesburg, Gauteng province, than in other provinces.^52^

Another explanation for the low prevalence of smoking might be in the selection of participants. Participants in Study Group I were recruited from the HIV clinic, and participants in Study Group II were recruited from RCTs. It may be the case that people participating in RCTs were more aware of health hazards or had a healthier lifestyle, associated with a lower smoking prevalence. A study, performed in 2011 in the same hospital as the current study, reported a smoking prevalence of 15% amongst PLWH, whilst validation by urine samples showed a prevalence of almost 31%.^20^

Subsequent analysis of only smokers, including both HIV-positive and -negative participants, showed no effect of HIV on pulmonary function. Although no effect of HIV infection was observed, age was a significant factor in all models and analyses. This result supports our conclusion that the study population was too young to observe a possible additional negative effect of HIV on pulmonary function in smokers.

A limitation of this study is that CD4 cell count was not available. CD4 is a marker of immune impairment, and immune dysregulation may affect lung function. In this study, no association between ART and pulmonary function was observed, whilst some studies suggest that specific ART-regimens may be more strongly associated with decreased pulmonary function than other regimens.^5,22,53,54^ Another limitation is that this is a single-centre study, and, therefore, the results might not be generalisable to different contexts. As all but four participants were black, the study results are only applicable to black South Africans. Furthermore, no follow-up data are available. To investigate the true effect of smoking on pulmonary function, investigation of longitudinal data is necessary.

Finally, we only had information on pack-years available for current smokers; this is a powerful measure to reliably reflect the intensity of smoking, especially in LMICs, where the number of cigarettes smoked might be correlated with household income so pack-years could possibly be a more suitable and accurate measure than current versus past smoking. The correlation coefficient *R* = 0.40 (*P* < 0.01) between age and pack-years shows that participants with higher age also had significantly higher pack-years. This result supports our hypothesis that smoking may very well have influence on pulmonary function; however, our cohort is too young to establish this relationship.

Strengths of this study include the inclusion of a large urban population, representative of the general HIV-positive population in an urban environment in a low-middle income setting. Besides, we present a complementary analysis on the effect of HIV, smoking and lung function by analysing the effect of smoking in HIV-positive people, as well as in people who smoke the effect of HIV on lung function.

In summary, in a relatively young population of HIV-positive people, the negative impact of smoking on lung function is not yet measurable, and in smokers, HIV does not seem to be a risk factor for additional pulmonary impairment. A history of TB is, however, a strong risk factor for impaired pulmonary function.

More research studies are needed to longitudinally investigate the influence of smoking on decreasing FEV1 and FEV1/FVC ratio in the aging HIV-positive population in sub-Saharan Africa. In the meantime, smoking should be discouraged on every occasion, given the high burden of TB, associations with other lung infections and expected persistent lung damage in this population.

## Appendix 1

### Elaboration on the Method section

#### Measurements

Study group I: For spirometry test, the KoKo® Sx 1000 hand-held machine was used. Two outcomes were obtained from the spirometry test: the forced expiratory volume in 1 second (FEV1) in litres (L) and the forced vital capacity (FVC) in litres.^31^ Each participant was asked to perform a spirometry test twice: once before and once after administering four doses of 100 μg salbutamol for bronchodilation. Each spirometry was repeated until three acceptable and reproducible attempts were obtained per participant, with a maximum of eight attempts per test. Acceptability and reproducibly were based on guidelines of the American Thoracic Society and European Respiratory Society.^31,32^ Participants with an abnormal spirometry test were referred to a pulmonologist for review. Definitions of an abnormal test were set up prior to start of the study (Table 1-A2).

A questionnaire was used to retrieve information on socio-demographics, history of lung and other diseases, and physical activity. The respiratory questionnaire was based on the St George’s Respiratory Questionnaire (SGRQ),^30^ which was aimed at retrieving information on respiratory complaints, smoking, and pulmonary exposure during work and at home.

Smoking was limited to tobacco products (cigarettes, cigars and pipes) and was classified into never versus ever (past and current) smokers. Race was defined as black or non-black. HIV status was based on test results from UTT or ART prescription. Time since HIV diagnosis was measured in months. Education was divided into two groups: less than secondary school completed and secondary school completed or higher. Work status was split into four groups: employed, unemployed, retired and student. Relationship status was dichotomised into stable relationship yes or no, with stable relationship being defined as married or in a self-defined stable relationship. Alcohol use was defined as ever or never. Time spent close to an open fire was divided into daily versus less than daily and was defined as fire time. The presence of a smoker in the household during childhood was defined as smoking household. Smoking inside the house during childhood was defined as smoking household indoor. Fireplace childhood was defined as the presence of an indoor fireplace in childhood home. Parental lung disease was defined as either one of the participant’s parents being diagnosed with a chronic pulmonary disease. Occupational hazard was dichotomised into ever versus never, and was defined as exposure to gases, chemical fumes or chemicals during work, working in a dusty job, or working in the farming or mining industry for more than a year. Breathing problems was classified into presence or absence of breathing or chest troubles or symptoms in the past three months.

Study group II: The measurements taken in Study II were identical to those in Study I. Comparable questionnaires were used to assess demographics, characteristics and respiratory profile of participants of both the study groups. The respiratory questionnaire was based on the SGRQ,^30^ the British Medical Research Council Respiratory Questionnaire,^33,34^ the ATS-DLD-78-A,^35^ the World Health Survey^36^ and questionnaires used in other publications.^37,38^ Physical examination was also performed. For spirometry, a Spida 5 handheld machine was used with similar procedures and outcome assessment to the KoKo® Sx 1000 handheld machine.

**TABLE 1-A1:** Results of multivariable analysis for post-bronchodilator (BD) forced expiratory volume in 1 second and the forced expiratory volume in 1 second/forced vital capacity ratio, of imputed data sets.

Variable	Model 1: without history of TB		Model 2: with history of TB	
	Post-BD FEV1 (β-coefficient†)	Post-BD FEV1/FVC (β-coefficient†)	Post-BD FEV1 (β-coefficient†)	Post-BD FEV1/FVC (β-coefficient†)
Smoking ever	< 0.001	0.004	0.001	0.004
Age (years)	–0.022**	–0.002**	–0.022**	–0.002**
Sex (male)	0.836**	–0.020**	0.847 **	–0.019*
BMI (kg/m^2^)	–0.008*	–0.001*	–0.008*	–0.001 *
Time since HIV diagnosis (months)	< 0.001	< 0.001	< 0.001	< 0.001
ART duration (months)	< –0.001	< –0.001	< –0.001	< –0.001
Occupational hazard‡	0.032	< 0.001	0.023	< 0.001
History of pneumonia	–0.132*	–0.023*	–0.090	–0.019
Smoke household indoor‡	–0.043	0.005	–0.035	0.006
Fireplace childhood‡	–0.105*	0.001	–0.108*	< 0.001
History of TB	-	-	–0.200**	–0.018**

BD, bronchodilator; BMI, body mass index; FEV1, forced expiratory volume in 1 second; FVC, forced vital capacity; TB, tuberculosis.

* , *p* < 0.05;

** , *p* < 0.01.

† , β-coefficients are the result of multivariable analysis and are unstandardised;

‡ , Definitions of variables can be found in Methods: Measurements.

**TABLE 2-A1:** Spirometry definitions.

Diagnosis	Criteria
Asthma	FEV1/FVC < 0.70, and pre- and post-bronchodilation FEV1 difference of 12% and 200 mL or more
COPD	FEV1/FVC < 0.70, and no pre- and post-bronchodilation FEV1 difference of 12% and 200 mL or more
Obstructive lung disease	FEV1/FVC < 0.70, and FEV1 < 80% of predicted value
Healthy lung function	FEV1/FVC > 0.70, and FEV1 or FEV1/FVC > 80% of predicted value

FEV1, forced expiratory volume in 1 second; FVC, forced vital capacity; COPD, chronic obstructive pulmonary disease.
